# Definitive Radiation Therapy for Older Adults With Localized Prostate Cancer

**DOI:** 10.7759/cureus.108367

**Published:** 2026-05-06

**Authors:** Francis M Wright, Dexiang Gao, Christopher L Geiger, Tyler P Robin

**Affiliations:** 1 Internal Medicine, University of Colorado School of Medicine, Aurora, USA; 2 Pediatrics, Biostatistics and Bioinformatics Shared Resource, University of Colorado Anschutz Medical Campus, Aurora, USA; 3 Medical Oncology, University of Colorado School of Medicine, Aurora, USA; 4 Radiation Oncology, University of Colorado School of Medicine, Aurora, USA

**Keywords:** aua score, biochemical recurrence, definitive radiation therapy, genitourinary complications, geriatric oncology, geriatrics, localized prostate cancer, moderate hypofractionation, psa screening, radiation side effects

## Abstract

Introduction

Individual health and life expectancy, rather than age, should drive management decisions for patients with prostate cancer. There is limited available data regarding treatment outcomes and tolerance for older adults where health status warranted treatment with curative intent. Herein, we report treatment outcomes for patients 80 years and older who received definitive radiation therapy (RT) for localized prostate cancer.

Methods

This retrospective study includes 41 patients who received RT for localized prostate cancer at the age of 80 years or older between 2004 and 2022. The median age was 83. Baseline details and outcomes, including treatment toxicity, symptom scores, biochemical recurrence (BR), distant metastases (DM), and overall survival (OS), were collected in a secure database and analyzed with Graphpad Prism (GraphPad Software, Boston, MA, USA) and R software (version 4.5; R Foundation for Statistical Computing, Vienna, Austria).

Results

No patients experienced adverse events that were Common Terminology Criteria for Adverse Events (CTCAE) grade 4 or higher. One patient (2.4%) experienced late CTCAE grade 3 proctitis, seven patients (17.1%) experienced acute CTCAE grade 2 genitourinary adverse events (GU AE), and five patients (12.2%) experienced late grade 2 GU AEs. Mean urinary symptom scores improved after RT (with the International Prostate Symptom Score (IPSS) decreasing from 12.1 pre-RT to 8.2 post-RT). The estimated four-year cumulative incidence of metastases was 6.7% (95% CI 0-16.1%). There were trends in outcomes based on risk group, but no statistically significant differences between intermediate risk cancer (IR) and high or very high-risk cancer (HR/VHR) regarding BF (15% vs. 20%, p=0.17), DM (0% vs. 10%, p=0.20), or OS (94% vs. 96%, p=0.38).

Conclusions

Definitive RT for patients 80 years or older was generally well tolerated and effective in this small cohort. These findings may indicate a favorable risk/benefit ratio for definitive prostate cancer treatment for select patients 80 years or older. Decision-making for prostate cancer treatment should involve a comprehensive approach, accounting for each individual patient’s life expectancy and overall health rather than avoidance of treatment based simply on age.

## Introduction

Radiation therapy (RT) is a definitive treatment option for localized prostate cancer, providing equivalent efficacy compared to radical prostatectomy (RP) [1]. Side effects of RT include gastrointestinal and genitourinary symptoms, which generally resolve over time [2]. Urinary incontinence and sexual dysfunction are less common in patients receiving RT relative to RP, although gastrointestinal symptoms are more common [1,2]. The National Comprehensive Cancer Network (NCCN) guidelines recommend treatment for patients with high-risk disease and a life expectancy of more than five years. Treatment is also recommended for patients with unfavorable intermediate-risk disease and a life expectancy of more than 10 years, and can be considered for those with a life expectancy of 5-10 years [3].

Although these guidelines are in place, prostate-specific antigen (PSA) screening is often discontinued for patients older than age 70, irrespective of life expectancy. Notably, in 2012, the United States Preventive Services Task Force (USPSTF) recommended against PSA screening at all ages. Following these recommendations, screening rates decreased, and the incidence of metastatic prostate cancer rose significantly [4,5]. The symptom burden of metastatic prostate cancer includes bone pain, pathologic fracture, and spinal cord compression, which are associated with reduced quality of life and survival [6,7]. In 2018, USPSTF reversed this recommendation, but continues to recommend against screening patients age 70 or older [8]. Importantly, according to the Social Security Actuarial Life Table, patients ages 70 to 76 without significant co-morbidities have an average life expectancy of more than 10 years [9], which is a population that the NCCN guidelines would recommend treatment for if they were found to have high-risk or unfavorable intermediate-risk disease [3]. The USPSTF recommendations against screening may be in place partly due to concern that treatment may not reduce mortality [10]. In addition to screening cessation, treatment is often discouraged for older patients due to the concern that risks outweigh benefits.

This study aims to challenge this assumption by investigating the risk-benefit ratio of definitive RT for patients 80 years or older. The primary objective is to characterize clinical outcomes following definitive RT in this older population. The secondary objective is to compare how outcomes vary across intermediate-risk and high-risk disease. Characterizing toxicity and survival following definitive RT contributes to a more nuanced understanding of outcomes for older patients who wish to pursue curative treatment for localized prostate cancer.

## Materials and methods

Patient selection

In this retrospective study, patients 80 years or older who received definitive RT for localized prostate cancer at a single institution between January 1, 2004, and December 31, 2022, were computationally and manually identified. Definitive RT was defined as cases receiving RT with curative intent. Patients with localized prostate cancer included cases with or without regional lymph node involvement. Patients with metastases in nonregional lymph nodes, bone metastases, or metastases at other sites at the time of RT initiation were not included. Those who received salvage RT, palliative RT for metastatic prostate cancer, or initiated RT at an age younger than 80 were excluded. Cases with insufficient documentation regarding androgen deprivation therapy (ADT), RT protocol, oncologic outcomes, and treatment toxicities were also excluded.

Data collection

Charts were manually reviewed by a resident (F.W.) and a board-certified radiation oncologist (T.R.) to confirm that outcomes were properly classified, toxicities were graded appropriately, RT modalities were accurately categorized, and inclusion criteria were met (n=41). Relevant details regarding demographics, clinical history, American Joint Committee on Cancer (AJCC) stage, RT, ADT, oncologic outcomes, International Prostate Symptom Score (IPSS), and treatment toxicities were manually extracted using retrospective chart review and stored in a secure database. The study protocol was approved by the Colorado Multiple Institutional Review Board.

Analysis

Variables were summarized using mean, median, and range for continuous variables. Life expectancy from the age that patients started RT was estimated using the most recent Social Security Administration (SSA) Actuarial Table and adjusted using the clinician’s assessment of the quartile of health, which can be found in the NCCN Guidelines for Older Adult Oncology [11]. Biochemical recurrence (BR), distant metastases (DM), and overall survival (OS) were assessed for the overall cohort and stratified by NCCN risk group (intermediate risk (IR) and high/very high risk (HR/VHR)). One patient with N1 disease was included with the HR/VHR group for comparative analysis of oncologic outcomes. BR was measured from the end of RT and defined as the first PSA at least 2.0 above nadir. Patients without BR were censored at the last follow-up. DM was measured from the end of RT to the date of DM or censored at the last follow-up without DM. OS was measured from the end of RT to the date of death, with survival censored at the date of last follow-up. BR and OS were summarized using the Kaplan-Meier product-limited approach. IR and HR/VHR groups were compared using log-rank tests. DM was summarized using the cumulative incidence function due to the presence of competing risk from death. Because of the small number of events (three metastases and three competing deaths) among the study patients, we summarized results descriptively using the non-parametric cumulative incidence function, treating death as a competing risk. Toxicity was stratified by organ system, Common Terminology Criteria for Adverse Events (CTCAE) grade, and time of onset. Acute side effects, both patient and physician-reported, were defined as onset within three months of RT completion, while late side effects occurred more than three months after RT completion. Baseline IPSS was measured prior to initiation of both RT and ADT. IPSS was measured at two additional time points (six months and 12 months) following completion of RT. Median follow-up was calculated using the reverse Kaplan-Meier method. Statistical analysis was performed using Graphpad Prism (GraphPad Software, Boston, MA, USA) and R software (version 4.5; R Foundation for Statistical Computing, Vienna, Austria). Statistical significance was defined as a p-value of ≤0.05.

## Results

Demographics and treatment

Of the 41 patients included in the analysis (Table 1), the median age was 83 (IQR: 81-85). The median Eastern Cooperative Oncology Group (ECOG) performance status was 0 (IQR, 0-2). Median life expectancy was 7.3 years (IQR: 5.9-10.5). Regarding NCCN risk groups, two patients (4.8%) had favorable IR cancer, 13 (31.7%) had unfavorable IR cancer, and 25 (60.9%) had HR or VHR cancer (Table 2). One patient (2.4%) had lymph node (LN) positive disease. Fourteen patients (34.1%) received conventional fractionation, 22 (53.7%) received moderate hypofractionation, and five (12.2%) received stereotactic body radiotherapy (SBRT). Four patients (9.8%) received elective nodal radiation. Ten patients (24.4%) received no ADT, 20 (48.8%) received three to six months, and 11 (26.8%) received 12+ months.

**Table 1:** Baseline demographics. ECOG: Eastern Cooperative Oncology Group Performance Status, IQR: interquartile range

Patient Characteristics	All (n=41)		Intermediate Risk (n=15)		High/Very High Risk (n=26)	
	Median	IQR	Median	IQR	Median	IQR
Age	83	81-85	82	81-82.5	83.5	82.0-85.0
ECOG	0	0-2	0	0-1	1	0-1
Life Expectancy	7.3	5.9-10.5	7.3	5.9-10.5	7.1	5.3-9.9

**Table 2:** Clinical information. NCCN: National Comprehensive Cancer Network, SBRT: stereotactic body radiation therapy, ADT: androgen deprivation therapy

NCCN Risk Group	Total, n (%)
Low or Very Low	0
Favorable Intermediate	2 (5)
Unfavorable Intermediate	13 (32)
High or Very High	25 (61)
Node-Positive	
Yes	1 (2)
No	40 (98)
Radiation	
Conventional Fractionation	14 (34)
Moderate Hypofractionation	22 (54)
SBRT	5 (12)
Lymph Nodes Treated	
Yes	4 (10)
No	37 (90)
ADT	
None	10 (24)
3-12 months	20 (49)
12+ months	11 (27)

Outcomes

**Figure 1:**
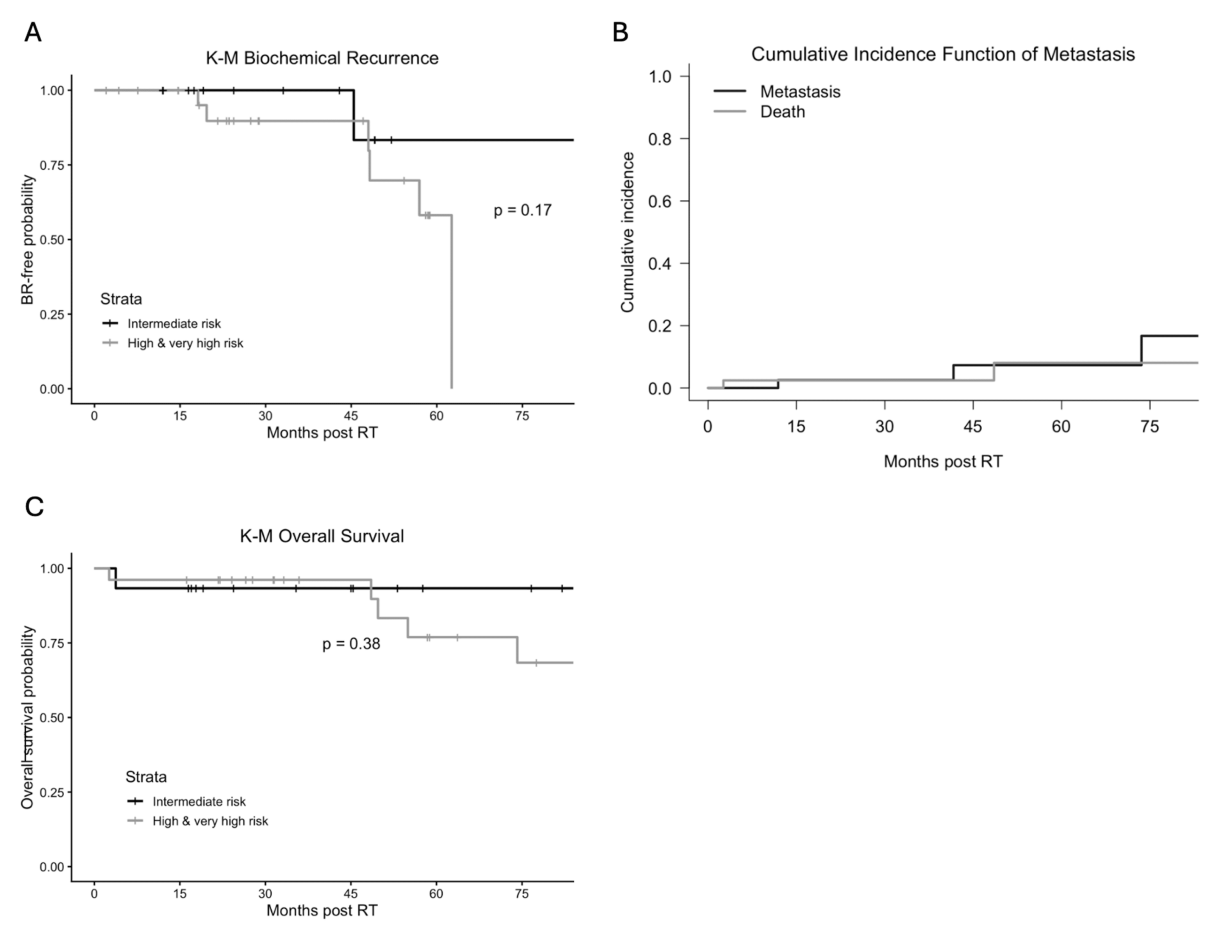
Kaplan-Meier estimation grouped by NCCN risk group (IR and HR/VHR). (A) Biochemical recurrence, measured from the end of RT, is defined as the first PSA at least 2.0 above nadir. (B) Distant metastases (DM), measured from the end of RT to the date of DM, or the last follow-up without DM. (C) Overall survival, measured from the end of RT to the date of death or last follow-up. Overall survival was estimated at four years from the end of RT. NCCN: National Comprehensive Cancer Network, RT: radiation therapy, IR: intermediate risk, HR/VHR: high/very high risk

Median follow-up was 57.6 months (95% CI 35.4-82.1) for all the study patients, 45.2 months (95% CI 24.4-88.6) for patients with IR, and 61.3 months (95% CI, 33.2-not reachable) for patients with HR/VHR. Seven patients experienced BR during the study follow-up time. All patients who died had experienced BR prior to death; therefore, death was not a competing risk for BR. Using the Kaplan-Meier method, the estimated four-year PSA failure rate for the overall cohort was 19% (95% CI 0-35%), with rates of 15% in the IR group and 20% in the HR/VHR group (p=0.17) (Figure 1).

Due to death (three events) as a competing risk for DM (three events), we used the Fine-Gary cumulative function to summarize the incidence. The estimated four-year cumulative incidence of metastasis was 6.7% (95% CI 0-16.1%). This result indicates that, within this small cohort, the absolute risk of developing metastasis remained low with treatment. We were unable to estimate DM for the IR and HR/VHR separately due to the small number of events.

For OS, the estimated four-year survival rate is 95.1% (95% CI 88.8-100%) for the overall cohort, and 94% and 96%, respectively (p=0.38) for the IR and the HR/VHR groups.

Toxicity

**Figure 2:**
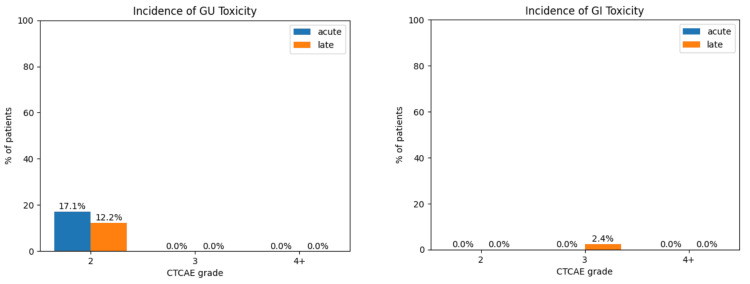
Toxicities stratified by CTCAE grade, onset (acute vs. late), and organ system involvement. Acute toxicity was defined as occurring within three months of RT completion. Late toxicity was defined as occurring after three months of RT completion. GU: genitourinary, GI: gastrointestinal, CTCAE: Common Terminology Criteria for Adverse Events, RT: radiation therapy

**Figure 3:**
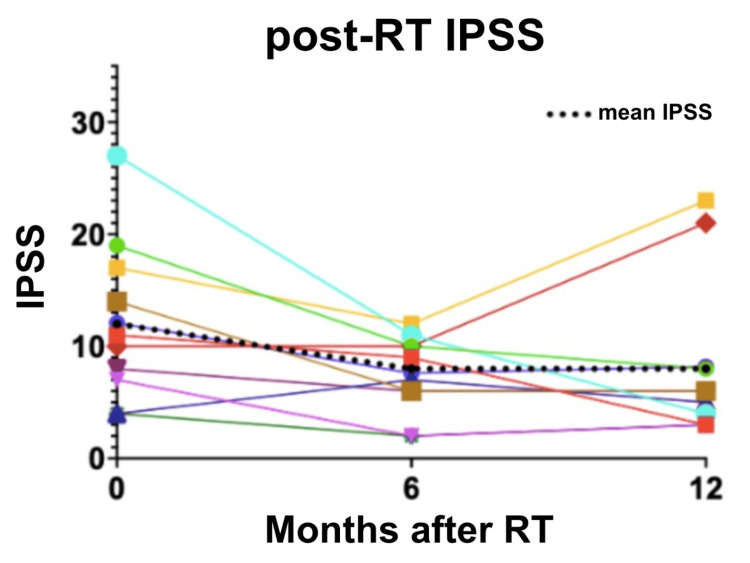
Spaghetti plot representing IPSS for each patient at three time points: 0, 6, and 12 months. IPSS: International Prostate Symptom Score, RT: radiation therapy

No patients experienced adverse events (AEs) that were CTCAE grade 4 or higher (Figure 2). One patient (2.4%) experienced a late grade 3 GI AE (proctitis). Seven patients (17.1%) experienced acute grade 2 GU AEs. Five patients (12.2%) experienced late grade 2 GU AE (urinary frequency, incontinence, urethral stricture). No patients experienced acute or late G3 or higher GU AEs. Mean IPSS prior to treatment was 12.1. Mean IPSS fell to 7.6 six months after RT and remained lower than pre-treatment, with a mean of 8.2 one year after RT (Figure 3). Eleven patients (27%) were included in the IPSS analysis, while the remaining were excluded for missing data.

## Discussion

This study characterized clinical outcomes and side effects for patients older than age 80 with localized prostate cancer who received definitive RT. Outcomes, including BR, DM, and OS, were similar to the general population of patients with prostate cancer, whose median age at diagnosis is 67 and median age at initiation of RT ranges from 67 to 71 [12-14]. Although no significant difference was found, there were worse trends in oncologic outcomes for patients with high-risk disease, consistent with previous studies for patients younger than age 80 [15-17]. These findings provide evidence that older patients who receive curative RT may have similar clinical outcomes compared to patients younger than age 80. These outcomes may be clinically relevant for patients older than age 80 with limited comorbidities who are interested in curative treatment for localized prostate cancer.

Side effects following RT occurred at similar rates to those seen in the general population, although gastrointestinal side effects were reported less often than in previous studies [18-20]. Side effects primarily involved the genitourinary system, with rare gastrointestinal toxicity. Gastrointestinal side effects are among the primary concerns for patients regarding RT toxicities, so the low frequency of gastrointestinal toxicity in this study group is notable. One patient (2.4%) suffered from grade 3 side effects, which is lower than observed in many studies in the general population [21], and there were no grade 4+ toxicities, indicating that RT may be a safe treatment modality for older patients.

Mean IPSS decreased after RT. Mean IPSS was lowest six months after RT, then rose slightly at 12 months, although the standard deviation was large at that time point. This pattern differs from previous studies that showed IPSS rising at six months before improving at 12 months [2]. Mean IPSS remained lower than pre-RT levels at 12-month follow-up, indicating potential symptomatic benefit in addition to curative therapy. Bladder outlet obstruction related to prostate cancer may improve with pelvic RT [22], so it is possible that malignant obstructive symptoms were more common in this group than in other studies looking at IPSS changes over time following RT. Notably, medications for lower urinary tract symptoms were not rigorously captured, limiting the interpretation of these findings in the context of concurrent symptomatic management. Additionally, initiation of ADT may have contributed to the observed improvement in IPSS scores. However, a small subgroup of patients did not receive ADT (n=10), limiting the ability to perform robust subgroup analysis.

It is important to highlight that older patients have been found to have a higher frequency of metastatic prostate cancer at presentation [23], which may be related to reduced PSA screening [8] and a higher prevalence of aggressive prostate cancer in this population [24]. Additionally, patients older than age 80 have a higher incidence of death from prostate cancer, despite higher death rates from competing causes [23]. With these trends in mind, the findings of this retrospective study may affect the risk-benefit calculation for older patients considering definitive RT. Larger, prospective studies are needed to validate these findings.

A limitation of this study is the small sample size (n=41), limiting the power to detect rare toxicities. Larger studies are indicated to continue surveillance for less common side effects in this population. A second limitation is that this is a single-institution study performed at an academic center, potentially limiting generalizability. Additionally, exclusion of patients without sufficient documentation may have biased against inclusion of patients who follow up out-of-state or at the Veterans Health Administration. Another limitation is the use of the most recent SSA actuarial table, which may overestimate life expectancy, as these values typically rise over time. Finally, this study did not have a control group, so outcomes and symptoms following RT could not be compared to patients who opted for active surveillance, other forms of curative treatment, focal therapies, or medical therapy alone.

## Conclusions

This retrospective study found that RT was well-tolerated in patients older than age 80, who may have similar outcomes compared to the general population. Gastrointestinal side effects were rare, and the mean IPSS improved after RT. These findings provide evidence that RT may be an ideal treatment modality for older patients seeking to cure localized prostate cancer.
